# Editorial: Repurposed Drugs as Immune-Modulators to Combat Infectious Diseases

**DOI:** 10.3389/fimmu.2022.848373

**Published:** 2022-02-02

**Authors:** Bibhuti B. Mishra, Makram Essafi, Ramandeep Singh, Shashank Gupta, Suraj P. Parihar

**Affiliations:** ^1^ Department of Immunology and Microbial Disease, Albany Medical College, Albany, NY, United States; ^2^ Laboratory of Transmission, Control and Immunobiology of Infection (LTCII), Pasteur Institute of Tunis, Tunis, Tunisia; ^3^ Translational Health Science and Technology Institute (THSTI), Faridabad, India; ^4^ Division of Intramural Research, National Heart, Lung and Blood Institute, National Institutes of Health, Bethesda, MD, United States; ^5^ Wellcome Centre for Infectious Disease Research in Africa (CIDRI-Africa) and Institute of Infectious Diseases and Molecular Medicine (IDM), University of Cape Town, Cape Town, South Africa

**Keywords:** repurpose approach, infectious disease, adjunctive therapies, immunomodulation, vaccine

Repurposed drugs offer efficient treatment options as monotherapy or adjunctive for diseases that have fewer or no therapeutic interventions. In this Research Topic, we collected new data on the potential of repurposed drugs against infectious diseases, including tuberculosis and COVID-19.

Tuberculosis (TB) is an infectious disease caused by *Mycobacterium tuberculosis (Mtb)*, a bacterium responsible for the maximum number of deaths before SARS-CoV-2. Given severe toxicity, low efficacy, and the duration (minimum six months) often affect patients’ adherence leading to the emergence of drug resistance. Generating new antibiotics to overcome resistance is a too costly and time-consuming process as evident with the development of new antibiotics Bedaquiline and Delamide, to treat drug-resistant TB, required approximately 20-30 years. In this Research Topic, Fatima et al. discussed the beneficial use of drugs such as sulfonamides, sulfanilamide, sulfadiazine, clofazimine, linezolid, amoxicillin, carbapenems, metformin, verapamil, fluoroquinolones, statins and NSAIDs repurposed to treat TB. This also included mechanisms of action with emphasis on their immunomodulatory effects on the host to attain both host- and pathogen-directed therapies for the potential synergistic effect as adjunctive TB therapies.

The success of *Mtb* has been related to its ability to manipulate the host effector mechanisms, including phagosome escape and/or maturation, autophagy, antigen presentation, and metabolic pathways. In this Research Topic, Nienaber et al. highlighted another aspect of HDT, which is based on the modulation of the TB-associated inflammatory response through the manipulation of lipid mediators metabolism. The authors summarized mainly preclinical studies, about the beneficial effects of Non-steroidal anti-inflammatory drugs (NSAIDs) and omega-3 long-chain polyunsaturated fatty acids (n-3 LCPUFA) on the outcome of TB treatment. By reducing the host exacerbated inflammation, the lipid manipulators contributed to the decrease of bacterial burden and ameliorated the treatment of the infection. In agreement, Hayford et al. showed that indeed co-administration of ibuprofen (short-term) and n-3 LCPUFA as an adjunct decreased mycobacterial loads and improved lung tissue pathology by decreasing pro-inflammatory cytokines in C3HeB/FeJ mice. These studies suggested that LCPUFA is a suitable candidate as an adjunct to frontline TB drugs. However, clinical trials are needed to confirm the benefit/safety of patients.

The balance between the timing and levels of pro- and anti-inflammatory responses plays a key role in the fate of *Mtb* infection. An excessive pro-inflammatory response may cause an enlargement of granuloma and tissue damage, which may prolong the length of TB treatment and permanently diminish the lung function of TB survivors. The review by Krug et al. provided comprehensive information on drugs that target inflammatory (corticosteroids, MMPs, PARP1, TNF antagonists) or anti-inflammatory (Tregs and MDSCs) pathways to control lung damage and bacterial growth in preclinical models. However, the beneficial/detrimental role of these drugs is dependent on the time of usage during the disease. Consequently, therapies that modulate this spectrum of immune responses at the appropriate time may have the potential to improve the treatment of TB or reduce the permanent lung damage after a microbiological cure. The repurposed drugs known to modulate such responses may improve the future of TB therapy, however, the adverse effect of drug-drug interaction and their bioavailability may be a limiting factor for such host-directed therapies.

The ideal host-directed therapeutics for TB should potentiate the antimycobacterial defenses while preventing excessive inflammation and tissue injury. While the conventional anti-TB therapy uses a combination of antibiotics to maximize clearance of *Mtb* by targeting the pathogen metabolism, ion channel blockers could enhance bacillary clearance by targeting both the pathogen and host immune responses. Ion channel blockers alter cell physiology by attenuating ion exchange across the cellular and subcellular membrane commonly used to treat diseases such as hypertension. It is notable that several FDA-approved ion channel blockers have shown promise at both restricting *Mtb in vitro* and attenuating inflammation *in vivo*. Additionally, some ion channel blockers have direct antimycobacterial activity. In this Research Topic, Mitini-Nkhoma et al. provided a review of the literature on the clinically approved ion channel blockers that demonstrated anti-tuberculosis activity in *Mtb*-infected macrophages and/or in the animal model proposed as potential HDTs against drug-sensitive and -resistant TB.

The lung macrophages are one of the major myeloid cells that *Mtb* colonizes to establish infection. *Mtb* infected macrophages are more permissive to generate HIV viral particles rendering HIV-TB coinfection very challenging to treat. Protease inhibitors (PIs), which targets the viral replication cycle, are in clinical use to treat HIV. However, their effectiveness to treat HIV-TB coinfection is of great research interest. *Mtb* inhibits macrophage phagosome maturation and downregulates the lysosomal hydrolases such as Cathepsin S and H. Chemical scaffolds that relieve this blockade have the potential to boost *Mtb* killing and limit HIV replication during coinfections. In this Research Topic, Pires et al. showed that Saquinavir (SQV), a PI sold as Invirase/Fortovase to treat patients with HIV, increases lysosomal Cathepsin S protease activity, which increases the killing of *Mtb* in HIV coinfected human macrophages. Interestingly, by yet unidentified mechanisms, SQV enhances the expression of the MHC II at the cell surface; increases T cell priming, proliferation, and IFN-γ production. This study establishes the potential of SQV as a potential candidate for HDT against TB. Although there are no experimental models to test the effectiveness of SQV in HIV-TB coinfections, further studies could offer more insights into this clinically approved drug as an HDT for TB.

HDT also has the potential to modulate host-immune responses which can improve the efficacy of BCG vaccine or frontline TB drugs. Bouzeyen et al. showed that co-administration of MK-2206, an inducer of apoptosis, enhanced the ability of BCG to impart protection against *Mtb* in mice and guinea pigs through multiple mechanisms; FOXO3 activation, enhanced BCG induced apoptosis, inhibition of macrophage IL-10 secretion, and increased BCG induced effector/memory T cells. Furthermore, the lung tissue damage in animals immunized with BCG/MK-2206 was reduced in comparison to BCG alone immunization. In addition to MK-2206, Berberine, a plant-derived natural compound, is historically used to treat diabetes and hypertension. Ozturk et al. showed that as an adjunct, it improved the efficacy of frontline TB drugs. The administration of berberine with isoniazid (INH) and rifampicin (RIF) increased *Mtb* killing in murine and human macrophages. This approach also decreased lymphoid, myeloid cells recruitment and production of inflammatory cytokine/chemokine such as CXCL-10, IL-1β, and CCL3 in the lungs of mice. The availability of toxicology data for these studies would reduce the development timeline to be evaluated in Phase II/III clinical trials.

Besides TB, Nontuberculous mycobacterial (NTM) infections present a serious challenge to clinical management due to several innate resistance mechanisms of mycobacteria. Additionally, the current NTM treatment regimen presents significant clinical side effects in patients. In this Research Topic, Crilly et al. proposed host-directed immune-modulatory therapies for treating *Mycobacterium avium* complex (MAC). The authors included and discussed pathways; autophagy and PD-1/PD-L1 as viable HDT targets. In addition to targeting excessive pathological inflammation using anti-TNF antibodies, the authors described compounds with broad activity like statins and metformin. Future research to develop MAC HDT presents major opportunities with significant challenges and better treatment options for NTM patients.

Sepsis represents a major clinical problem and cause of death for patients in intensive care units worldwide. Among the pro-inflammatory mediators known to drive the pathogenesis of sepsis, plasma IL-1β level is associated with poor prognosis. Experimental models of LPS induced septic shock have shown the role of NLRP3 inflammasome activation in regulating IL-1β production and driving acute inflammatory pathologies. Both genetic and pharmacological inhibition of NLRP3 inflammasome ameliorates the acute inflammation and tissue damage caused during sepsis. In this Research Topic, Luo et al. investigated the role of the FDA approved Fat mass and obesity-related protein (FTO) inhibitor “Entacapone” as a potential therapeutic for sepsis. FTO is the primary *N* ^6^-methyladenosine demethylase, well studied in obesity, however, the role in inflammatory diseases remains unclear. The knockdown (siRNA) and inhibition (entacapone) of FTO, hindered macrophage activation, tissue damage and improved survival in LPS-induced endotoxic shock in mice. Mechanistically, the ablation of FTO inhibited the NLRP3 inflammasome regulated IL-1β production through FOXO1/NF-kB signaling in macrophages. Therefore, targeting FTO offers potential treatment options for life-threatening sepsis and similar pathologies.

In 2020, the COVID-19 pandemic has ravaged healthcare systems around the world. Patients infected with SARS-CoV-2 present diverse inflammatory sequelae with the progression of the disease. The terminally ill patients present severe lung inflammation, cytokine storm, and extensive lung damage that ultimately affects the air exchange and leads to mortality. While antivirals may be beneficial at the early stages of COVID-19, anti-inflammatory drugs such as dexamethasone have been effective in limiting the severity of disease by targeting the overwhelming inflammatory response and cytokine storm. Burrage et al. discussed the potential and clinical data from the use of various immunomodulatory agents targeting cytokines (IL-1, IL-6, TNF), inflammasome (NLRP3 inhibitor, colchicine), systemic inflammation (corticosteroids). While there are multiple clinical trials are underway, this review will help design randomized double-blinded clinical trials to find evidence-based therapies in the context of this global pandemic. For instance, Caracciolo et al. showed the use of Canakinumab (anti-IL-1β monoclonal antibody) in the case of an 85-year-old male, treated for the severe form of SARS-CoV-2 infection for compassionate use. The condition of the patient deteriorated with acute respiratory distress syndrome, cardiac and renal failure after 25 days of hospitalization, was intubated. Subsequently, diuresis recovered, and condition improved: high IL-6 levels and NK cells expressing CD56^bright^ (associated with cytokine release) were significantly reduced giving rise to NK CD56^dim^. Unfortunately, the patient did not survive after day 58 owing to persistent SARS-CoV-2 infection and pulmonary bacterial superinfection. Therefore, canakinumab rescued a high-risk, very elderly patient, from multiorgan damage complicating COVID-19. It may represent a useful treatment in severe cases; however, further studies are warranted.

## Author Contributions

All authors contributed to the article and approved the submitted version.

## Funding

The editorial is based in part on work supported by AI148239-01A1 NIAID/NIH (BBM), BT/IN/Indo-Tunisia/01/2014 (ME and RS) and 203135Z/16/Z (SPP).

## Conflict of Interest

The authors declare that the research was conducted in the absence of any commercial or financial relationships that could be construed as a potential conflict of interest.

## Publisher’s Note

All claims expressed in this article are solely those of the authors and do not necessarily represent those of their affiliated organizations, or those of the publisher, the editors and the reviewers. Any product that may be evaluated in this article, or claim that may be made by its manufacturer, is not guaranteed or endorsed by the publisher.

